# Cannabinoids for treating inflammatory bowel diseases: where are we and where do we go?

**DOI:** 10.1080/17474124.2017.1292851

**Published:** 2017-02-16

**Authors:** Carina Hasenoehrl, Martin Storr, Rudolf Schicho

**Affiliations:** ^a^ Institute of Experimental and Clinical Pharmacology, Medical University of Graz, Graz, Austria; ^b^ Department of Medicine, Ludwig-Maximilians University, Munich, Germany; ^c^ Zentrum für Endoskopie, Starnberg, Germany

**Keywords:** Cannabinoids, Crohn’s disease, dronabinol, inflammatory bowel disease, medical marijuana, nabilone, nabiximols, ulcerative colitis, Cannabis

## Abstract

**Introduction**: Fifty years after the discovery of Δ^9^-tetrahydrocannabinol (THC) as the psychoactive component of Cannabis, we are assessing the possibility of translating this herb into clinical treatment of inflammatory bowel diseases (IBDs). Here, a discussion on the problems associated with a potential treatment is given. From first surveys and small clinical studies in patients with IBD we have learned that Cannabis is frequently used to alleviate diarrhea, abdominal pain, and loss of appetite. Single ingredients from Cannabis, such as THC and cannabidiol, commonly described as cannabinoids, are responsible for these effects. Synthetic cannabinoid receptor agonists are also termed cannabinoids, some of which, like dronabinol and nabilone, are already available with a narcotic prescription.

**Areas covered**: Recent data on the effects of Cannabis/cannabinoids in experimental models of IBD and in clinical trials with IBD patients have been reviewed using a PubMed database search. A short background on the endocannabinoid system is also provided.

**Expert commentary**: Cannabinoids could be helpful for certain symptoms of IBD, but there is still a lack of clinical studies to prove efficacy, tolerability and safety of cannabinoid-based medication for IBD patients, leaving medical professionals without evidence and guidelines.

## 1. Introduction

Inflammatory bowel diseases (IBDs), i.e. Crohn’s disease (CD) and ulcerative colitis (UC), are chronic inflammatory conditions of the gastrointestinal (GI) tract with increasing prevalence in Westernized countries [1]. Although their etiology is still unknown, these diseases are thought to comprise misdirected attacks of the immune system against gut microbiota or their products [2]. Defects in the epithelial barrier function and in mucosal wound healing are of paramount importance in the progression of IBD [1,2]. The endocannabinoid system (ECS) has been recognized to play an important role in the maintenance of gut homeostasis since it quickly responds to disturbances by *de novo* synthesis of its effector molecules and is, therefore, of particular interest in the management of IBD [3].

**Figure 1.**
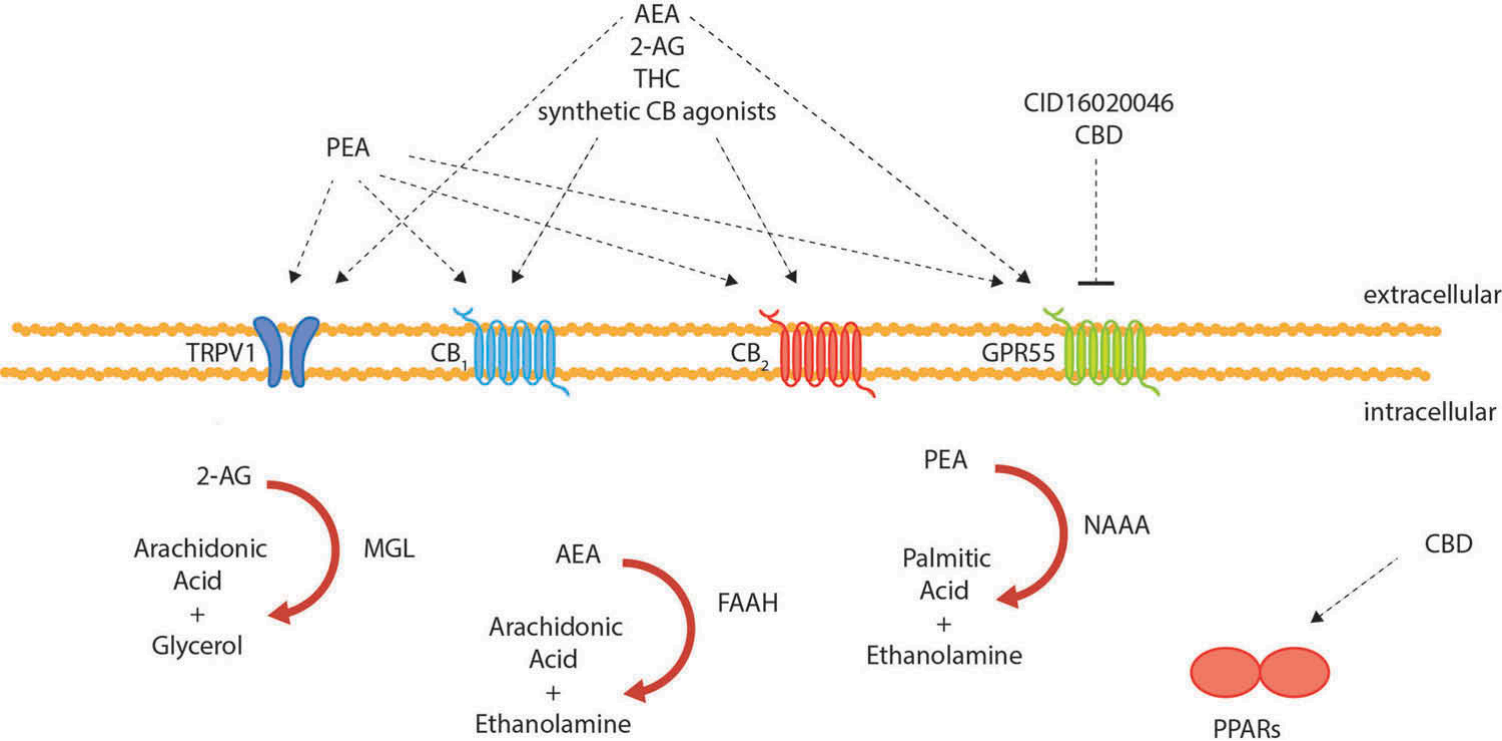
A schematic overview of cannabinoid receptors, cannabinoid-responsive non-cannabinoid receptors, their ligands and degrading enzymes of the endocannabinoid system as described in murine IBD. 2-AG, 2-arachidonoylglycerol; AEA, anandamide; CB, cannabinoid receptor; CBD, cannabidiol; FAAH, fatty acid amide hydrolase; GPR55, G protein-coupled receptor 55; NAAA, N-acylethanolamine-hydrolyzing acid amidase; PEA, palmitoylethanolamide; PPARs, peroxisome proliferator-activated nuclear receptors; THC, Δ^9^-tetrahydrocannabinol; TRPV1, transient receptor potential of vanilloid-type 1.

The ECS consists of lipid mediators, so-called endocannabinoids, their synthesizing and degrading enzymes, and of G protein-coupled cannabinoid receptors (CBs) that mediate the endocannabinoid effects (Figure 1). Components of the ECS have been found to be expressed throughout the GI tract and have been reviewed in detail elsewhere [4,5]. Briefly, the ECS has been described to comprise two CBs, i.e. cannabinoid receptor 1 (CB_1_) and 2 (CB_2_). CBs can be activated by a variety of synthetic or plant-derived cannabinoids, as well as by the endocannabinoids anandamide (arachidonoylethanolamine [AEA]) and 2-arachidonoylglycerol (2-AG). Synthesizing enzymes include *N*-acyl phosphatidylethanolamine phospholipase D (NAPE-PLD) for AEA and diacylglycerol lipase for 2-AG, respectively.

Degradation of AEA is facilitated mainly by fatty acid amide hydrolase (FAAH), whereas 2-AG is degraded mostly by monoacylglycerol lipase (MGL or MAGL). However, the notion of the ECS being a confined physiological entity with CB receptors in its center has been challenged by the discovery of receptors responsive to cannabinoids other than CB_1/2_, such as G protein-coupled receptor 55 (GPR55), transient receptor potential of vanilloid-type 1 (TRPV1), and peroxisome proliferator-activated nuclear receptors (PPARs) [6]. Furthermore, it has been found that the ECS does not only consist of the aforementioned biosynthetic and degrading components, but also in fact shares many enzymes with other pathways, e.g. cyclooxygenase-2 which oxidizes AEA and thus provides a link between the ECS and prostaglandin synthesis [7]. While the complexity of the interactions of all molecules involved in (endo-) cannabinoid signaling represents a large obstacle in understanding the endocannabinoids’ role in (patho-)physiology, research on the ‘expanded ECS’ or ‘endocannabinoidome’ [7] may very well open doors for new treatment options. After all, it is desirable to discover cannabinoid based drugs that exert their actions without causing psychotropic effects that arise from activation of central CB_1_ receptors.

## 2. The ECS as a therapeutic target in IBD

To investigate the role of cannabinoids in IBD, mostly animal models that rely on chemically induced mucosal inflammation are used. Dextran sulfate sodium (DSS)-induced colitis, for instance, causes the influx of macrophages, neutrophils, and a Th2-mediated immune response [8–10], whereas trinitrobenzene sulfonic acid (TNBS)-induced colitis is more dominated by a Th1-response [8,10]. Analysis of expression levels of ECS components in inflamed rodent colonic tissue revealed enhanced cannabinoid signaling under inflammatory conditions as compared to healthy tissue. Thus, CB_1_ and CB_2_ receptors, as well as AEA, were found to be upregulated in experimental IBD models [11,12]. Upregulation of AEA, however, was only found in certain layers of the colon (e.g. in the submucosa but not the mucosa) [11]. On the other hand, the AEA-degrading enzyme FAAH was expressed to a lesser extent in the initial stage of colitis but returned to control levels as the disease progressed [13]. Pharmacological strategies to enhance endocannabinoid levels in the inflamed colon of rodents through inhibition of the degrading enzymes FAAH or MGL, respectively, ameliorated the inflammation [14,15]. Accordingly, it has been reported that activation of CB_1_ or CB_2_ with synthetic agonists protected from colitis [12,16] and that treatment with Δ^9^-tetrahydrocannabinol (THC), the main psychoactive constituent of *Cannabis sativa*, reduced TNBS-induced inflammation as well as myeloperoxidase (MPO) activity and motility disturbances in the in rat colon [17]. These findings prompted the investigation of other nonpsychoactive components of *Cannabis* in IBD models. It has been shown that cannabidiol (CBD), a cannabinoid with very low affinity for CB_1_ and CB_2_, has protective effects in murine colitis as observed by a reduction of colon injury, inducible nitric oxide synthase expression, reactive oxygen species production, MPO activity, and tumor necrosis factor alpha (TNF-α) levels [17–21]. CBD has also been reported to inhibit FAAH activity [22] and could thus alter endocannabinoid levels. Other non-psychotropic cannabinoids, shown to be beneficial in colitis models, include the plant cannabinoid cannabigerol [23] and the synthetic atypical cannabinoid O-1602, which has been shown to inhibit neutrophil recruitment [24]. Cannabigerol reduced nitric oxide production in macrophages and this effect was modulated by the CB_2_ receptors [23]. While the molecular targets of cannabigerol and O-1602 have not been fully elucidated yet, extensive evidence exists that CBD exerts its functions, at least partly, through PPARs [21]. CBD has also been reported to act as an antagonist to GPR55, a receptor that plays a crucial role in intestinal inflammation [25]. Another molecule of interest in IBD is palmitoylethanolamide (PEA), a structural relative of anandamide that acts via multiple targets including CB_1_, CB_2_, GPR55, PPARα, and TRPV1 and that has been reported to reduce inflammation and intestinal permeability in mice [26–28]. With regard to the ECS, beneficial effects of PEA in experimental IBD involved an increase in colonic CB_1_ receptor expression and activation of CB_2_ and GPR55 [26]. Another plant cannabinoid with anti-inflammatory properties in murine colitis is cannabichromene [29] which inhibited endocannabinoid inactivation [30].

Taken together, a huge amount of preclinical data strongly support the ECS as a therapeutic target in IBD (as previously reviewed by Refs. [3,31–33]).

### 2.1. Components of the ECS are differentially expressed in human IBD

The altered regulation of the ECS in IBD patients has been addressed in various reports with rather contradictory outcomes (summarized in Table 1). Although AEA levels were found to be increased in UC patients (*n* = 8) [11], some studies reported an overall reduced AEA signaling in IBD patients, as observed through decreased activity and/or levels of the synthesizing enzyme NAPE-PLD [34,35], as well as through increased activity of the degrading enzyme FAAH [34,35], and through reduced levels of AEA [34]. Enhanced CB_2_ immunoreactivity has been observed in the colonic epithelium and some cells of the inflammatory cell infiltrate in CD and UC specimens, suggesting that CB_2_ might be a relevant target for IBD treatment [34–36]. In fact, activation of CB_2_ has shown protection in experimental models of colitis [16,37]. Also in a human colonic explant model, where colitis-like damage was induced with pro-inflammatory cytokines, activation of CB_2_ led to reduced damage of mucosal crypts and the epithelial lining [38].

**Table 1.** Differential expression of ECS components in human IBD compared to controls as described in the literature.

		IBD		
ECS component		UC	CD	References
Receptors	CB_1_	No change, downregulation, or upregulation	Upregulation	[26–28]
	CB_2_	Upregulation		[27,30]
		Upregulation (protein) or no change (mRNA, protein)		[26,28]
Ligands	AEA	Upregulation		[10]
		Downregulation		[26]
	2-AG	No change		[10,26]
Synthesizing enzymes	NAPE-PLD	Downregulation or reduced activity		[26,27]
	DAGL	Upregulation		[27]
Degrading enzymes	FAAH	No change (epithelium) or upregulation (immune cells) or increased activity		[26,27]
	MGL	Upregulation		[27]

CB_1_: cannabinoid receptor 1; CB_2_: cannabinoid receptor 2; AEA: anandamide; 2-AG: 2-arachidonoylglycerol; ECS: endocannabinoid system; IBD: inflammatory bowel disease; NAPE-PLD: *N*-acyl phosphatidylethanolamine phospholipase D; DAGL: diacylglycerol lipase; FAAH: fatty acid amide hydrolase; MGL: monoacylglycerol lipase.

Furthermore, treatment with methanandamide, a non-hydrolysable analog of AEA, reduced interferon-γ and TNF-α secretion from cultured biopsy specimens and from lamina propria cells isolated from IBD specimens [34].

### 2.2. Could Cannabis be used as a treatment for IBD?

Thousands of years ago, *Cannabis* was traditionally used for the treatment of inflammation of the gut. Nowadays, the use of medicinal *Cannabis* is being legalized in a growing number of countries but clinical studies on the effects of *Cannabis* in IBD are scarce. Questionnaires conducted in Canada, USA, and Israel so far revealed that patients commonly use the drug as a self-medication to relieve IBD-related symptoms, including abdominal pain, diarrhea, and loss of appetite [39–43]. A retrospective observational study on 30 patients with CD found that 21 subjects significantly benefited from *Cannabis* consumption as seen through a significant reduction of the average Harvey Bradshaw Index as well as through a reduced need for other medication [44]. A prospective pilot study with 13 IBD patients, who were instructed to inhale *Cannabis* when they were in pain, concluded that the treatment significantly improved the patients’ quality of life [42]. Finally, a small randomized placebo-controlled clinical trial suggested beneficial effects of *Cannabis* treatment in CD patients [45]. In this study, 11 patients received *Cannabis* cigarettes containing 115 mg THC twice daily, while the placebo group (*n* = 10) received *Cannabis* flowers that were devoid of THC. The duration of the study was 8 weeks with an additional wash out phase of 2 weeks. Because of the small sample sizes, a statistical difference in remission (a Crohn’s disease and activity index [CDAI] < 150) between treatment (5/11) and placebo group (1/10) was not achieved. A clinical response (a reduction in CDAI > 100), however, was observed in 10/11 subjects in the treatment group (4/10 in the placebo group). Additionally, in the *Cannabis* group, three patients were weaned from steroids and two from opiates. Patients of the treatment group further reported an increase in quality of life (as assessed by SF-36). A difference in objective markers of inflammation, i.e. C-reactive protein, however, was not observed between the two groups [45]. A summary of clinical studies on the benefit of *Cannabis* medication in IBD conducted to date is given in Table 2. It should be kept in mind, however, that most of these clinical studies are statistically underpowered and also lack methodological quality. Another critical point is the use of the right placebo in these studies as central effects of *Cannabis*/cannabinoids are hard to conceal.

**Table 2.** An overview of clinical studies on Cannabis treatment in IBD patients.

	Patient number (*n*)					
Study type	UC	CD	Treatment	Investigated parameters	Findings	Reference
Questionnaire	100	191	–	Cannabis use, SIBDQ	33% of UC and 50% of CD patients used Cannabis for symptom relief	[31]
Questionnaire	63	231	–	Cannabis use, subjective assessment, users vs. nonusers	17.6% of patients used Cannabis for symptom relief, surgery prediction	[33]
Retrospective observational study	–	30	Cannabis use (unspecified)	HBI, need for surgery and hospitalization	HBI reduced from 14 ± 6.7 to 7 ± 4.7	[36]
Prospective study	2	11	Cannabis use for 3 months (not standardized)	HBI, partial Mayo score	HBI reduced from 11.36 ± 3.17 to 5.72 ± 2.68	[34]
Prospective study	–	21	115 mg THC twice daily or placebo for 8 weeks	CDAI, life quality (SF-36)	Remission (5/11), CDAI reduction (10/11), increased life quality	[37]
Prospective survey	102	177	–	Cannabis use	16.4% of patients used Cannabis for symptom relief	[32]
Prospective survey	18	35	–	Cannabis use, SIBDQ in young adults	45% of patients (18–21 years old) used Cannabis for symptom relief	[35]

HBI: Harvey Bradshaw index; IBD: inflammatory bowel disease; THC: Δ^9^-tetrahydrocannabinol; CDAI: Crohn’s disease and activity index; SIBDQ: short-inflammatory bowel disease questionnaire.

### 2.3. CBD for the treatment of IBD?

As outlined above, several preclinical studies have indicated that CBD is protective in intestinal inflammation [17–20]. A study in an lipopolysaccharide-induced model suggested that CBD, which is known to act as GPR55 antagonist [46], inhibits GI inflammation by controlling the inflammatory response and the activation of enteric glial cells [21]. Parts of the beneficial effects by CBD were mediated via PPARγ raising the possibility that GPR55 could have been involved in the beneficial effect [21]. Furthermore, CBD could be supportive in maintaining a healthy intestinal barrier. In a CaCo-2 cell monolayer model stimulated by EDTA, CBD recovered the intestinal barrier in a concentration and CB_1_-dependent manner [47]. The results of a clinical trial on CBD in IBD (ClinicalTrials.gov ID NCT01037322), however, have not been published so far. The anti-inflammatory potential of CBD has been also recently reviewed elsewhere [48].

## 3. Conclusion

Experimental evidence gathered from preclinical IBD models and conducted in rodents point to a strong potential of the ECS components to serve as drug targets in inflammatory diseases of the intestine. Data suggest a homeostatic role of the ECS in the gut. Accordingly, it is believed that the enhancement of endocannabinoid signaling, as observed through the increased levels of endocannabinoids and their receptors, and the decrease in endocannabinoid degrading enzymes, is a response to disturbances of the homeostatic system and is aimed at restoring the balance. This is further supported by the finding that the manipulation of the ECS toward a further increase of endocannabinoid signaling is protective against IBD. On the other hand, analysis of biopsies from UC and CD patients paints a rather complex picture in terms of differential expression of ECS components. Most likely, owing to the small sample sizes in the studies, a conclusion on the meaning of this has not yet been reached. Most evidence points toward an involvement of CB_1_ and also CB_2_ receptors, especially with regard to immune cell recruitment. Further research in this direction, preferably on human IBD material, such as explants, cultured biopsies, etc. is highly warranted.

## 4. Expert commentary

IBDs pose a high burden on patients and health-care systems alike. Current therapy includes anti-inflammatory agents like aminosalicylic acid, immunomodulators, steroids, and biological agents, such as anti-TNF-α antibodies and vedolizumab (an anti-α4β7 integrin antibody that specifically targets adhesion and migration of leukocytes to the GI tract) [49]. Vedolizumab has shown its ability and effectiveness in the induction therapy of IBD, in particular, in patient’s refractory to anti-TNF-α antibody treatment [50]. Biologicals are first-line alternative treatment options in severe IBD and are powerful tools to change the course of the disease [49,51]. However, they come at the prize, as in the case of anti-TNF-α antibodies, of causing severe adverse effects, such as infections, malignancies, and injection/infusion reactions [52]. For Vedolizumab, cases of arthritis and sarcoiliitis have been recently reported [53]. Conventional treatment is often little effective leaving many patients dissatisfied [54]. Because of failing standard therapy, willingness and desire of IBD patients to use complementary and alternative medicine (CAM) are understandably high and an alternative treatment with *Cannabis* is, therefore, often sought.

Not surprisingly, a New Zealand Survey of 1370 patients with IBD revealed that 44.1% of IBD patients self-medicated with CAM, including *Cannabis* [55]. Additionally, a large population-based study, using data from the NHANES database (National Center for Health Statistics) showed that IBD patients had a higher incidence (67.3% vs. 60.0%) and an earlier onset in age (15.7 vs. 19.6 years) for *Cannabis* use as compared to control subjects [56]. Judging from these data, *Cannabis* has long been used by IBD patients as a form of ‘self-treatment’ to control their symptoms. In the US prospective cohort survey of IBD patients, Ravikoff Allegretti reported that half of the patients, who had never used *Cannabis* before, expressed their interest in *Cannabis* for treatment, in particular, against abdominal pain [40].

The broad interest for alternative medicine often puts medical professionals in a situation in which patients express their wish to use *Cannabis* medicinally, but at the same time, they also face legal and administrative boundaries for its prescription. Unlike in Canada, Israel, and some states in the United States, ‘medical marijuana’ is not legally available in most European countries (only after seeking permission from the government) although cannabinoid-based drugs, such as nabiximols (Sativex®), dronabinol, and nabilone, can be obtained (Table 3). Prescription of these drugs has to follow strict indications or may be given off-label with a narcotic prescription, a situation many physicians are reluctant to deal with.

**Table 3.** Currently available cannabinoids for human treatment.

**Dronabinol** ((–)-Δ^9^-trans-tetrahydrocannabinol)
Application: oral
Trade name: Marinol®
**Nabilone** (THC analog)
Application: oral
Trade name: Cesamet®
**Nabiximols** (combination of THC and cannabidiol)
Application: sublingual spray
Trade name: Sativex®
**Medical Marijuana** (dried leaves and buds from *Cannabis*) Application: oral, by inhalation, topical
Traded as ‘Medical Marijuana’ or ‘Medical Cannabis’

THC: Δ^9^-tetrahydrocannabinol.

### 4.1. Basic versus clinical research with Cannabis/cannabinoids

A surge of preclinical data on cellular mechanisms of CB receptors and other components of the ECS have recently come forward. However, more information on *in vivo* mechanisms of cannabinoids and the ECS in inflammatory diseases like IBD is necessary, despite existing reports [19–21,23,25,26,57]. Although we can assume that anti-inflammatory actions of cannabinoids employ peripherally as well as centrally located CB receptors [58,59], a broader picture on how cannabinoids improve severity of inflammation in IBD is needed. Most likely, the ECS concerts a holistic anti-inflammatory response linking endocannabinoids with CB receptors and non-CB_1_/CB_2_ cannabinoid-responsive receptors, such as GPR55 [25] and PPARα [26]. Thus, the ECS exerts profound effects on the GI-immune system, intestinal barrier, motility, and brain areas that control gut homeostasis [58].

As to clinical research, data on human trials with *Cannabis*/cannabinoids in IBD unfortunately lag behind. The medical practitioner has no evidence or guidelines when confronted with questions whether and how to use *Cannabis*/cannabinoids for the treatment of IBD. Exactly this is of critical importance as the general public is becoming increasingly interested in trying medical treatment with *Cannabis*. With the availability of medical marijuana and the lack of guidelines for treatment, people may tend to apply *Cannabis* with the help of a whole range of unprofessional advice and views, an extremely undesirable situation.

### 4.2. Clinical evidence, efficacy, and safety of cannabinoid-based treatment in IBD

Evidence that *Cannabis*/cannabinoids provide benefit for IBD patients is still sparse. Apart from anecdotal reports and some small clinical trials [42,44,45], as well as a handful of questionnaires [39–41,43], not much is known regarding a benefit for IBD patients by *Cannabis*. After reviewing the small amount of literature available on *Cannabis*/cannabinoids and human IBD, some indication can be seen for the use of *Cannabis* against abdominal pain, nausea, and loss of appetite in CD but treatment should only be considered after careful assessment of risks and after failure of standard medication. With regard to UC, indication that *Cannabis* may provide benefit for patients mainly derives from anecdotes and surveys [39–41]. Altogether, clinical trials are urgently warranted to determine the efficacy of *Cannabis*/cannabinoids in IBD and to draw a comprehensive conclusion. *Cannabis*/cannabinoids have been considered generally safe as a short-time medication for adults but as a meta-review recently pointed out, there is also a risk of serious adverse events in short-term treatment [60]. High odds for side effects include psychiatric, nervous system, and hepatobiliary disorders; however, short-term treatment mostly causes not only dry mouth, dizziness, somnolence, euphoria, but also hallucinations [60]. These psychotropic-adverse effects are caused by activation of CB_1_ receptors in the brain. A possibility to circumvent this obstacle could be the use of cannabinoids that do not enter the blood–brain barrier or that only act peripherally.

### 4.3. Risks of long-term treatment with Cannabis/cannabinoids in IBD

Many questions arise when considering long-term treatment with *Cannabis*/cannabinoids which is likely necessary in diseases like IBD. Long-term treatment is a strong caveat in adolescent people, given the young age of 20 years for the onset of CD [61]. Prolonged use of *Cannabis* has shown neurological changes in adolescents including a decrease in gray matter volume in certain brain areas [62]. This aspect needs close attention because a new survey in young adults with IBD (from 18 to 21 years) reported that 70% of these patients did not discuss marijuana use with their gastroenterologist and only half of them had knowledge of possible adverse effects [43]. A further aspect to be considered when using *Cannabis* for medical treatment is pregnancy. A recent study suggests an increase in stillbirth by long-term use of *Cannabis* [63]. Since IBD is frequently accompanied by comorbidities, such as cardiovascular disease [64], patients taking *Cannabis* should be aware of the risk of myocardial infarction and stroke [65].

Long-term treatment with *Cannabis*/cannabinoids may also have pronounced effects on gut permeability. While THC and CBD have shown permeability-preserving and CB_1_-dependent properties in CaCo-2 monolayers, the opposite was observed for the endocannabinoids AEA and 2-AG [47,66]. Treatment with FAAH inhibitors that raise endocannabinoid levels may therefore unfavorably affect gut leakiness that is associated with IBD. To minimize risks of adverse effects by cannabinoids, which are caused by activation of CB_1_ receptors in the brain, the CB_2_ receptor may be an alternative and valuable target to treat IBD. Unlike with the CB_1_ receptor, activation of CB_2_ does not lead to psychotropic effects. In addition, experimental data point to a crucial role of CB_2_ in the protection against colitis [16,37]. New compounds have been recently described as selective CB_2_ agonists capable of ameliorating inflammation during DSS colitis [67] but it remains to be evaluated whether CB_2_ agonists are also anti-inflammatory in human IBD.

### 4.4. Application of Cannabis/cannabinoids in IBD

Medical marijuana is preferably applied via inhalation. Bioavailability levels of THC average at about 30% [68]. Oral THC has been shown to be effective in chemotherapy-induced nausea at a dose of 5–15 mg/m^2^ [69]. Most variabilities in THC bioavailability are due to the individual itself and the different content of THC in marijuana plants, a general problem of phytotherapy [68]. These obstacles can be circumvented by using purified ingredients of *Cannabis* or combinations of purified ingredients, as in nabiximols (THC and CBD), which is applied as a sublingual spray. Each milliliter of nabiximols contains 27 mg THC and 25 mg CBD and a meta-review summarized that the most common maximum dose was 8 sprays/3 h or 48 sprays/24 h in studies of spasticity, pain, nausea, and vomiting [60]. A possibility for IBD patients to undergo treatment with *Cannabis*/cannabinoids may be by intrarectal (IR) application. Preclinical studies in mice have shown an anti-inflammatory effect after IR application of CBD [18]. The IR route could reduce first pass effects and would allow cannabinoids to act also locally at receptors of the ECS which is highly represented in the bowel mucosa [35]. Doses of CBD have been evaluated in clinical studies of psychosis and anxiety and range between 200 and 800 mg/d [60]. Most common doses for dronabinol and nabilone were 5–30 mg/d (1–2 doses/d) and 2 mg 2× d, respectively, in studies of spasticity, pain, nausea, and vomiting [60].

## 5. Five-year view

Since the beginning of cannabinoid research, which is marked by the cloning of the CB_1_ receptor [70], the field has rapidly expanded, in particular, during the past decade. In 2011, the search term ‘cannabinoid’ yielded over 7000 hits in PubMed, the US National Library of Medicine, while this number has soared above 20,000 in 2016. However, for the gastroenterologist, it is sobering to realize that not even a 100 hits pop up when using the terms ‘cannabinoid’ and ‘inflammatory bowel disease'.

With increasing interest of the public and the attention of funding agencies, this will hopefully change in the years to come. By implementation of clinical trials on *Cannabis*/cannabinoids in IBD and the search for potential mechanisms of action in humans, the topic will certainly shift into the focus of the research community. On the preclinical side, we will gain more information on relevant basic mechanisms of cannabinoids in experimental IBD, for instance, on the role of CB receptors in the interaction of the microbiome with the gut epithelial barrier [71]. Although preliminary data on the efficacy of CBD in human CD do not appear to be promising [72], CBD still may find a way into treatment of IBD, after carefully evaluating dose and mode of application. From experimental data, FAAH inhibitors could hold high promise in the treatment of IBD [14,73,74]. However, human trials have failed to provide efficacy of these inhibitors [75]. In addition, the tragic incidence during the phase I trial for the FAAH inhibitor BIA 10-2474 has now hampered the translation of these drugs into humans, although up to that incident, other FAAH inhibitors were generally safe in human trials [76,77]. Here, we may have to come back to preclinical research to better understand the (patho-)physiological role of FAAH, and the mechanisms and caveats of these inhibitors.

The future will shed more light on drugs that directly target enzymes of the ECS to raise levels of endocannabinoids and other lipid compounds, such as PEA. Thus, exogenous PEA has been shown to reduce severity of experimental colitis [26]. Levels of PEA can be also increased endogenously in the colon via inhibition of N-acylethanolamine-hydrolyzing acid amidase leading to an improvement of experimental inflammation of the colon [27]. Certainly, inhibitors of MGL that raise endogenous levels of 2-AG will be in the focus during the years to come. IBD patients could benefit from such drugs through their antinociceptive and anti-inflammatory potential [78]. On the clinical side, we expect to see an increase in clinical studies (observational, controlled, and prospective) evaluating the efficacy of cannabinoid compounds in IBD. Some of the key questions are not answered yet, e.g. which patients would benefit the most from a treatment with cannabinoids and what the appropriate dosages and modes of application are. Other questions include the risks of long-term treatment and which group of patients should be excluded from treatment with cannabinoids.

There is an almost unanimous request in the current literature to conduct larger clinical trials to find out whether treatment with *Cannabis*/cannabinoids could provide benefit to the IBD patient. *Cannabis* is still rated as an illegal drug in most countries. However, because of the high public interest in cannabinoid-based medication, health-care providers, political institutions, and funding agencies hopefully will promote these kinds of studies by providing financial support and by easing legal barriers that still put patients and physicians in a semi-illegal position. For the researcher, one thing is certain: apart from THC and CBD, other anti-inflammatory compounds of the *Cannabis* plant are still waiting to be explored offering a vast and promising research area. However, physicians need definite proof that *Cannabis*/cannabinoids are effective in human IBD as well as guidelines for potential use. Fifty years after the discovery of THC by Raphael Mechoulam [79], the time is more than ripe.

## Key issues

- The GI tract accommodates all the relevant components of the endocannabinoid system and is therefore highly amenable to treatment with *Cannabi*s/cannabinoids
- Preclinical data in mouse models show that *Cannabis*/cannabinoids reduce the severity of colitis and relax intestinal hypercontractility through cannabinoid receptors and other components of the endocannabinoid system
- *Cannabis*/cannabinoids not only act via classical cannabinoid receptors but also via GPR55, TRPV1, and PPAR receptors
- To circumvent side effects of cannabinoid compounds, levels of endocannabinoids can be raised by inhibitors of endocannabinoid-degrading enzymes, such as FAAH and MGL. These enzymes are important drug targets in IBD.
- Translation of preclinical data on cannabinoids in experimental IBD into humans has been insufficiently investigated warranting large clinical trials
- *Cannabis* may be helpful in Crohn’s disease to ease abdominal pain but should be only considered after failure of standard medication and assessment of risks
- Short term treatment with cannabinoids may cause common adverse effects like dry mouth, fatigue and dizziness but also serious adverse effects occur.
- Until clinical trials do not provide evidence that IBD patients will benefit from a treatment with *Cannabis*/cannabinoids and before the questions of safety and tolerability are not solved, treatment with *Cannabis*/cannabinoids for IBD should not be recommended
